# PyAMARES, an Open-Source Python Library for Fitting Magnetic Resonance Spectroscopy Data

**DOI:** 10.3390/diagnostics14232668

**Published:** 2024-11-27

**Authors:** Jia Xu, Michael Vaeggemose, Rolf F. Schulte, Baolian Yang, Chu-Yu Lee, Christoffer Laustsen, Vincent A. Magnotta

**Affiliations:** 1 Department of Radiology, University of Iowa, Iowa City, IA 52242, USA; chu-yu-lee@uiowa.edu; 2 GE HealthCare, 2605 Brondby, Denmark; michael.vaeggemose@gehealthcare.com; 3 MR Research Centre, Department of Clinical Medicine, Aarhus University, 8000 Aarhus, Denmark; cl@clin.au.dk; 4 GE HealthCare, Oskar-Schlemmer-Str. 11, 80807 Munich, Germany; rolf.schulte@gehealthcare.com; 5 GE HealthCare, Waukesha, WI 53188, USA; baolian.yang@gehealthcare.com; 6 Department of Psychiatry, University of Iowa, Iowa City, IA 52242, USA; 7 Department of Biomedical Engineering, University of Iowa, Iowa City, IA 52242, USA

**Keywords:** magnetic resonance spectroscopy, AMARES, MRS quantification, open-source software, metabolite quantification, X-nuclei, Python, ^31^P MRS, dynamic MRS

## Abstract

**Background/Objectives**: Magnetic resonance spectroscopy (MRS) is a valuable tool for studying metabolic processes in vivo. While numerous quantification methods exist, the advanced method for accurate, robust, and efficient spectral fitting (AMARES) is among the most used. This study introduces pyAMARES, an open-source Python implementation of AMARES, addressing the need for a flexible, user-friendly, and versatile MRS quantification tool within the Python ecosystem. **Methods**: PyAMARES was developed as a Python library, implementing the AMARES algorithm with additional features such as multiprocessing capabilities and customizable objective functions. The software was validated against established AMARES implementations (OXSA and jMRUI) using both simulated and in vivo MRS data. Monte Carlo simulations were conducted to assess robustness and accuracy across various signal-to-noise ratios and parameter perturbations. **Results**: PyAMARES utilizes spreadsheet-based prior knowledge and fitting parameter settings, enhancing flexibility and ease of use. It demonstrated comparable performance to existing software in terms of accuracy, precision, and computational efficiency. In addition to conventional AMARES fitting, pyAMARES supports fitting without prior knowledge, frequency-selective AMARES, and metabolite residual removal from mobile macromolecule (MM) spectra. Utilizing multiple CPU cores significantly enhances the performance of pyAMARES. **Conclusions**: PyAMARES offers a robust, flexible, and user-friendly solution for MRS quantification within the Python ecosystem. Its open-source nature, comprehensive documentation, and integration with popular data science tools enhance reproducibility and collaboration in MRS research. PyAMARES bridges the gap between traditional MRS fitting methods and modern machine learning frameworks, potentially accelerating advancements in metabolic studies and clinical applications.

## 1. Introduction

Magnetic resonance spectroscopy (MRS) is a valuable tool for studying metabolic processes within biological systems in vivo. Numerous quantification methods have been developed for both time- and frequency-domain MRS, including recent deep-learning algorithms [1,2,3]. Among these, accurate, robust, and efficient spectral fitting (AMARES) stands out as one of the most widely cited time-domain fitting techniques, using prior knowledge of metabolite spectral lineshapes to obtain reliable results.

AMARES models the MRS signal as a sum of exponentially damped sinusoids. It uses parameters such as chemical shift, linewidth, amplitude, phase, and spectral lineshape, which can be constrained by prior knowledge [4,5]. This knowledge includes initial parameters, parameter ranges, and relationships between different peaks and can be readily obtained from published literature. Peaks outside the region of interest can be filtered out, and parameters without prior knowledge can be fitted [1,6].

In contrast, frequency-domain fitting methods like LCModel require all metabolites to be modeled as basis set spectra [2,7]. While this approach reduces the number of parameters to fit, it requires additional effort to obtain basis set spectra through experiments or numerical simulations. Moreover, frequency-domain fitting strategies typically require well-phased absorptive spectra. AMARES circumvents the sometimes subjective and complicated phasing procedure, making it particularly effective for analyzing data with distorted phases due to long receiver dead times. LCModel and AMARES have been compared directly and proven to be comparable, each with its own advantages [8,9,10,11,12,13,14]. However, AMARES is often the preferred method for quantifying X-nuclei MRS data, such as ^13^C and ^31^P MRS [11,15,16], where spectra typically exhibit fewer peaks and less J-coupling compared to ^1^H MRS. Also, AMARES can be used for post-processing, such as removing residuals from short-echo ^1^H MRS data measuring mobile macromolecules (MM) [17,18,19]. Recently, the introduction of pure-shift NMR techniques that simplify J-coupling split multiplets to singlets shows promise in facilitating AMARES fitting of ^1^H MRS [20].

Currently, the implementation of AMARES is limited to a few platforms. The most popular MRS quantification software, jMRUI (v 7.0), provides a range of spectral processing and quantification methods but requires the 32-bit Java environment to operate on Microsoft Windows and GNU/Linux systems [21]. Meanwhile, OXSA [5] and the older SPID [22] software are based on MATLAB (MathWorks, Natick, MA, USA, https://www.mathworks.com/, accessed on 6 October 2024). Python features a strong ecosystem due to its extensive libraries, robust community, and versatility in a wide range of applications, from web development to artificial intelligence (AI). Although there are numerous Python-based MRS and NMR software packages, such as FSL-MRS (https://git.fmrib.ox.ac.uk/fsl/fsl_mrs, accessed on 6 October 2024) [23], Vespa (https://vespa-mrs.github.io/vespa.io/, accessed on 6 October 2024) [24], SUSPECT (https://github.com/openmrslab/suspect, accessed on 6 October 2024) [25], and nmrglue (v 0.10) [26], none have implemented the AMARES algorithm.

In response, we developed pyAMARES (https://github.com/HawkMRS/pyAMARES, accessed on 6 October 2024), an open-source Python implementation of AMARES. This package provides the MRS community with a flexible, robust, and user-friendly platform that facilitates advanced MRS quantification and integrates seamlessly with machine learning pipelines. Unlike its Java and MATLAB counterparts, pyAMARES employs Excel and CSV spreadsheets to store initial values and constraints for fitting, making the creation and editing of prior knowledge datasets much easier. Built within the Python ecosystem, pyAMARES is easy to install, inherently cross-platform, and can work either as a standard-alone application or together with other Python software as an imported library, making it readily integrable into users’ existing processing pipelines.

## 2. Materials and Methods

### 2.1. Software Design

#### 2.1.1. Workflow

**Figure 1:**
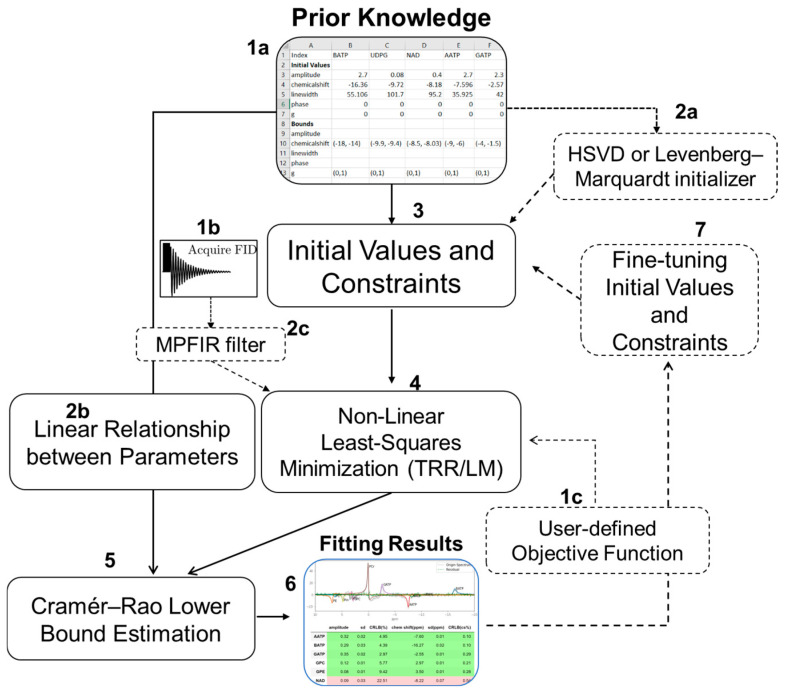
Flowchart of pyAMARES. The workflow starts with importing prior knowledge from spreadsheets (1a) and loading the FID signal (1b) to establish initial values and constraints for fitting (3). If the initial parameters are far from the actual values, users can optionally employ Hankel singular value decomposition (HSVD) or Levenberg–Marquardt (LM) initializers to optimize these starting values (2a). The FID signal can be processed directly or optionally filtered using MPFIR to focus on specific spectral regions (2b). The non-linear least-squares minimization (4) using either trust region reflective (TRR) or LM, with either default or user-defined objective functions (1c). The fitting process can be iterative—the output can be fine-tuned and used as initial parameters for subsequent iterations (7). The Cramér–Rao lower bound (CRLB) estimation (5) integrates information from both the fitting results and the linear relationships between parameters (2b). These relationships include constraints like fixed amplitude ratios or chemical shift differences between multiplet peaks. The final output (6) includes fitted parameters, their uncertainties (CRLB), and signal-to-noise ratios. Solid arrows indicate the main workflow, while dashed arrows and boxes represent optional processing steps.

PyAMARES fits MRS data to the model function $y_{n}={\hat{y}}_{n}+e_{n}=\sum_{k=1}^{K}a_{k}e^{j\varphi_{k}}e^{\left(-d_{k}\left(1-g_{k}+g_{k}t_{n}\right)t_{n}\right)}e^{j2\pi f_{k}t_{n}},n=0,1,\ldots ,N-1$, where n is the number of points. The parameters a_k_ (amplitude), f_k_ (frequency), d_k_ (damping factor), φ_k_ (phase), and g_k_ (lineshape) can be fit or fixed by pyAMARES as needed. Figure 1 shows the flow chart of pyAMARES. First, the prior knowledge of metabolites to quantify is provided in an Excel or CSV spreadsheet and converted into initial values and constraints for performing minimization with the lmfit library. If no prior knowledge dataset is provided, a Hankel singular value decomposition (HSVD) data initializer can be optionally invoked to generate the initial parameters. For better fitting results, an optional Levenberg–Marquardt (LM) or HSVD data initializer can be used to optimize the initial parameters. When necessary, the initialized fitting parameters or fitting results can be edited and improved for another round of fitting (Figure S1). Although AMARES is a time-domain fitting strategy, pyAMARES supports frequency-selective AMARES by either using an MPFIR [22] filter to extract the region-of-interest from the FID or a user-defined objective function that only minimizes the selected region. The Cramér–Rao lower bound (CRLB) is estimated from both the fitting results and the linear relationship between parameters provided by the prior knowledge [5,27]. The percentage of the CRLB of each parameter is calculated and added to the fitting results as a percentage.

#### 2.1.2. Prior Knowledge Spreadsheet

- Prior knowledge Dataset Format

Table 1 shows an example prior knowledge dataset for the ^31^P MRS of human brains at 7T, based on parameters from Ren et al. [28]. The spreadsheet is divided into initial values (upper half) and constraints (lower half). Parameters for each peak, such as relative concentration, chemical shifts, linewidth, phase, and lineshape (g), are defined in columns. Constraints are set using brackets for ranges or single values for fixed parameters; for instance, (−180, 180) specifies a range between −180 and 180, while (0,) indicates a minimum constraint of zero with no maximum constraint, and a single value like 0 indicates the parameter is fixed at 0. Amplitude and g are unitless; chemical shift is in parts per million (ppm), linewidth in hertz (Hz), and phase in degrees.

- Setting J-coupling Splitting for Multiplets

For naming peaks, especially multiplets, a primary peak name is suffixed with numeric identifiers to designate subpeaks—e.g., the triplet for β-ATP is labeled as BATP, BATP2, and BATP3. Numbers are not permitted in other peak names because they are reserved exclusively for labeling multiplets. Constraints for multiplets can use mathematical expressions to relate subpeak parameters. For example, phase and linewidth (LW) can be linked to the main peak, and subpeak chemical shifts can be adjusted relative to the main peak using J-coupling constants, such as BATP-15Hz, indicating a shift of 15 Hz less than β-ATP. Amplitude ratios are similarly constrained, with subpeaks having ratios defined relative to the main peak, like BATP/2 for β-ATP’s triplet.

The prior knowledge spreadsheet is parsed from left to right, requiring peaks that constrain others to be placed to the left of those they influence. For instance, BATP must precede BATP2 and BATP3 in the spreadsheet. Additionally, comments can be incorporated into the spreadsheet, marked by a starting #.

- Spreadsheets for Fitting Results

The fitting results are provided in two tables, *result_multiplet* and *result_sum*. The *result_multiplet* includes fitting results for all peaks, including sub-peaks in J-coupled multiplets, and *result_sum* combines all subpeaks in the multiplets by summing their areas. A colored version of the *result_sum* table, where the CRLB of peak amplitudes is smaller than 20%, is highlighted in green and can be accessed through *simple_df*. The signal-to-noise ratio (SNR) of each peak is reported as the fitted amplitude per one standard deviation (SD) of the noise [29].

### 2.2. Validation and Testing

The validation of pyAMARES is performed by comparing the fitting results to established software OXSA (v 2.0) and jMRUI (v 7.0) using an identical prior knowledge dataset.

A series of 500 singlet spectra are simulated with low, medium, and high SNR according to the AMARES model function. These spectra are generated with both fixed and perturbed spectral parameters, with Gaussian noise added to achieve various SNR levels. The performance of pyAMARES is evaluated against OXSA by comparing the fitted amplitudes, chemical shifts, and CRLBs across different SNR ranges.

Multi-peak spectra are simulated similarly using in vivo human brain ^31^P MRS at 7T reported in [28], with both fixed and perturbed parameters. The Monte Carlo simulations of in vivo human brain ^31^P MRS are conducted to assess the software’s capability in handling complex spectral patterns that typically appear under physiological conditions. The accuracy and precision of pyAMARES are quantified using relative bias, CRLB, and Pearson’s correlation coefficients compared to OXSA results.

To validate pyAMARES’s capability to model residual metabolite signals in the MM spectrum, we analyzed a short echo time ^1^H MR spectrum acquired at 9.4T, obtained from the supplementary material of Simicic et al. [17]. The raw data, provided in the jMRUI format, was read using the spec2nii Python library that is part of FSL-MRS [23]. The prior knowledge of metabolite signals was converted from the jMRUI format to a pyAMARES-compatible CSV file. Two methods were employed to align the spectrum: (1) shifting the FID signal so that the water peak was positioned at 4.7 ppm, and (2) adjusting the prior knowledge by offsetting the peak positions relative to the carrier frequency and the center of the readout. In both methods, the first 20 points of the FID were weighted using a quarter-sine wave function to match the weighting applied in jMRUI.

PyAMARES fits the data with the Levenberg–Marquardt (LM) algorithm for initialization, followed by a trust region reflective algorithm (TRR) fitting algorithm. The metabolite-free MM spectrum was obtained by subtracting the fitted metabolite signals from the original spectrum. For comparison, the residual spectrum generated by jMRUI was weighted using the same quarter-sine wave function and shifted to align the water peak at 4.7 ppm. The pyAMARES fitting results were compared to those obtained from jMRUI by examining the fitted amplitudes, linewidths, and CRLBs. The metabolite-free MM spectra produced by both methods were visually assessed for similarity.

### 2.3. X-Nuclei MRS Acquisition

#### 2.3.1. Deuterium Metabolic Imaging

Deuterium MR spectroscopic imaging (DMI) was performed on a 3T MRI scanner with a modified gradient noise filter (MR750, GE HealthCare, Waukesha, WI, USA) using a 27-cm dual-tuned (^1^H/^2^H) quadrature Tx/Rx volume coil (PulseTeq, Chobham, UK). A 3D MRSI sequence with density-weighted spiral readout was used [30]; matrix size = 10 × 10 × 10, FOV = 24 × 24 × 24 cm^3^, TR = 155.8 ms, flip angle = 70°, and NEX = 4. Data were acquired with 700 spectral points and a bandwidth of 5000 Hz, with a total scan time of 17:25 min.

#### 2.3.2. Xenon Imaging

Xenon images of porcine lungs were performed on a 3T MRI scanner (MR750, GE HealthCare, Waukesha, WI, USA) using a ^129^Xe transmit-receive quadrature vest coil (Clinical MR Solutions, Brookfield, WI, USA) tuned to 35.3 MHz. A 3D Cartesian MRSI sequence with spherical k-space sampling was used; matrix size = 28 × 28 × 6, field-of-view (FOV) = 40 × 40 × 20 cm^3^, number of excitations (NEX) = 2416. The sequence employed a spectrally-tailored RF pulse (duration = 0.6 ms, partial self-refocusing) designed to excite the dissolved and gas phases with flip angles of 10° and 0.1° and passbands of 500 Hz and 200 Hz, respectively [31]. The repetition time (TR) is 7.4 ms, resulting in a total acquisition time of 18 s, suitable for a single breath-hold in pigs. Data were acquired with 88 samples at a bandwidth of 20 kHz and zero-filled to 256, yielding a spectral resolution of 78 Hz (2.21 ppm).

#### 2.3.3. Dynamic ^31^P MRS

Phosphorus (^31^P) dynamic spectra of the tibialis anterior muscle were acquired on a 3T MRI scanner (MR750, GE HealthCare, Waukesha, WI, USA) using a ^31^P/1H dual-tune transmit-receive surface coil (RAPID Biomedical GmbH, Rimpar, Germany) tuned to 51.72 MHz. An unlocalized MRS sequence was used to acquire the dynamic spectra during rest, exercise, and recovery periods; TR = 2000 ms, flip angle = 90°, repetitions = 360, total acquisition time of 12 min. Data were acquired with 1024 samples at a bandwidth of 5 kHz, corresponding to a spectral resolution of 5 Hz (0.09 ppm).

## 3. Results

We present comprehensive Monte Carlo simulations evaluating pyAMARES’ performance with various datasets, along with examples that showcase pyAMARES’ various capabilities, including fitting MRS data with and without prior knowledge, selective-frequency fitting, modeling metabolite residuals in MM spectra, and multiprocessing fitting. These examples demonstrate pyAMARES’ flexibility and robustness in handling diverse MRS data analysis tasks.

### 3.1. Examples of In Vivo X-Nuclei (^31^P, ^129^Xe, and ^2^H) MRS Fitting

**Figure 2:**
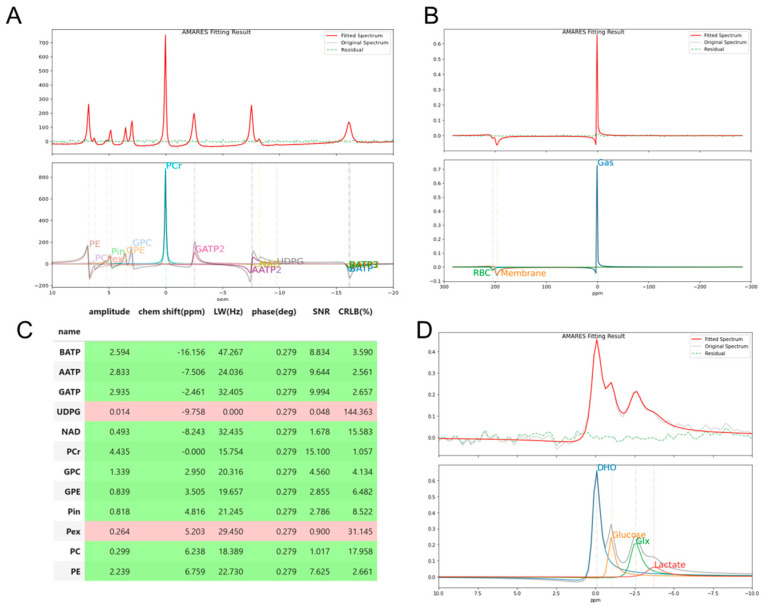
PyAMARES plotting outputs. The default output figure from the *plotAMARES* function shows the fit of (**A**) an in vivo brain ^31^P MRS spectrum acquired at 7T [34], (**B**) a voxel of hyperpolarized ^129^Xe MRSI acquired from healthy porcine lungs at 3T, and (**D**) a voxel of in vivo brain ^2^H 3D MRSI spectra acquired at 3T. In (**A**,**B**,**D**), the top panels display the original spectrum (gray), the fitted spectrum (red), and the residual (green dash), with individual fitted components shown in the bottom panels. Panel (**A**) is shown with phase correction applied (*ifphase = True* for the *plotAMARES* function) for display purposes, while (**B**,**D**) are not phased. The prior knowledge for the fitting (**A**) is in Table 1. The fitting results for ^31^P MRS (**A**), including metabolite concentrations and their respective Cramér–Rao lower bounds (CRLBs), are presented in (**C**), where green grows indicate reliable fits with CRLB < 20% and red rows indicate less reliable fits. The fitting results of (**B**,**D**) are shown in Figure S2. Abbreviations: RBC, red blood cells; DHO, deuterated water; Glx, combined signals of glutamate and glutamine; PCr: phosphocreatine; PE: phosphoethenolamine; GPE: glycerophosphoethanolamine; GPC: glycerophosphocholine; Pi: inorganic phosphate; NAD, nicotinamide adenine dinucleotide; UDPG, uridine diphosphoglucose.

Figure 2 and Figure S2 show the standard outputs of pyAMARES. For each spectrum (Figure 2A,B,D), the upper panel displays the original spectrum, fitted spectrum, residuals, and independent fitted components. Although AMARES is a time-domain fitting strategy, the *plotAMARES* function of pyAMARES performs a Fourier transform on the original and fitted FID signals and optionally applies phase correction when needed (Figure 2A). Figure 2C shows the simple HTML output spreadsheet of the fitting results of ^31^P MRS, with each row representing a metabolite. Metabolites whose peak amplitude CRLBs are below a customized threshold (default 20%) are colored green, indicating reliable fits. Otherwise, the corresponding metabolites are colored red. In the *resultpd* Pandas *dataframe* [32] of the fitting result, the multiplet peaks are shown as a single peak, while the amplitude of individual singlet peaks is available in the *result_multiplet* dataframe. The prior knowledge dataset and the corresponding HTML output for spreadsheets for ^129^Xe, and ^2^H can be seen in Figure S2. It should be noted that in the ^129^Xe fitting (Figure 2B and Figure S2A,C), the lineshapes for gas and red blood cells (RBC) are set to be Lorentzian (g = 0), while the lineshape for membrane is fit as a Voigt (initial value: g = 0.1) to account for its structural heterogeneity [33].

### 3.2. Validation of pyAMARES Using Monte-Carlo Simulation

**Figure 3:**
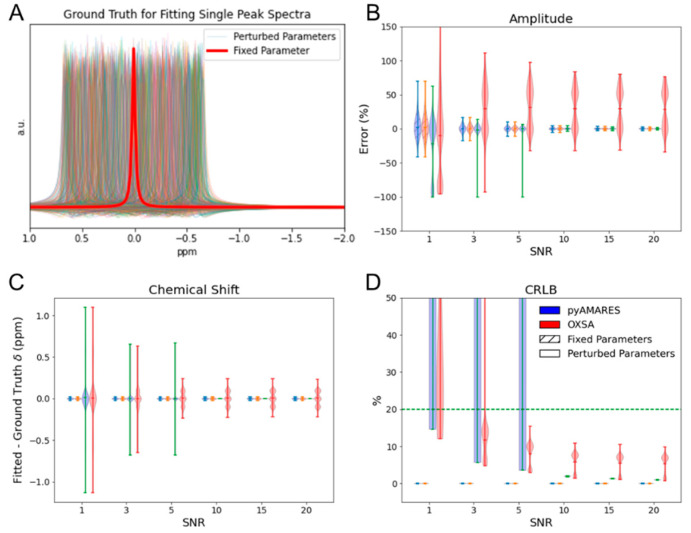
Comparison of Monte Carlo simulated single-peak spectra fitting using OXSA and pyAMARES. (**A**) Ground truth for spectra simulation with fixed (red) and 3000 perturbed (various colors) parameters. Gaussian noise is omitted for clarity. (**B**) Relative bias of fitted amplitude compared to ground truth at different SNR levels. (**C**) Bias of fitted chemical shift compared to ground truth at different SNRs. (**D**) CRLB of fitted amplitude at each SNR, with the 20% threshold indicated by a green dashed line. In (**B**–**D**), blue and red represent pyAMARES and OXSA fitted results, respectively; solid patterns indicate results from spectra simulated with perturbed parameters, while hatched patterns show results from spectra simulated with fixed parameters.

To evaluate the performance of pyAMARES against OXSA in single-peak spectral fitting, we conducted Monte Carlo simulations with both fixed and perturbed parameters (Figure 3A). Gaussian noise was added to the simulated spectra to achieve various signal-to-noise ratios (SNRs). The relative bias of fitted amplitude (Figure 3B), the bias of fitted chemical shift (Figure 3C), and the CRLB of fitted amplitude (Figure 3D) were assessed at different SNR levels. In simulations with fixed parameters, pyAMARES and OXSA performed similarly across all SNR levels. When SNR ≤ 5, OXSA showed less chemical shift bias and higher CRLBs compared to pyAMARES (Figure 3C,D). However, for spectra with perturbed parameters, pyAMARES demonstrated better accuracy and precision, particularly at SNR > 5. The relative bias of fitted amplitude and the bias of fitted chemical shift were lower for pyAMARES than for OXSA (Figure 3B,C, solid lines). Moreover, the CRLB of fitted amplitude for pyAMARES remained below the 20% threshold (green dashed line in Figure 3D) at lower SNR levels, while OXSA exceeded this threshold, suggesting more reliable quantification by pyAMARES. These results highlight the robustness of pyAMARES in handling single-peak spectra with perturbed parameters and its potential for improved quantification accuracy and precision when SNR ≥ 10.

**Figure 4:**
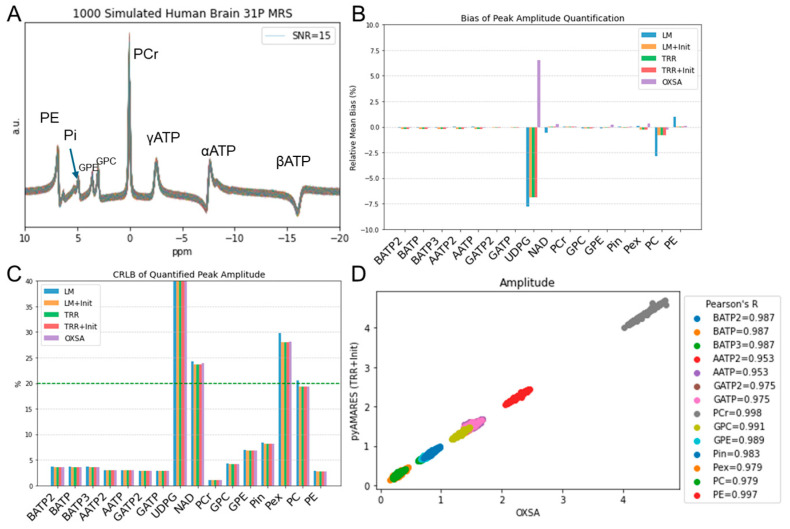
Comparison of Monte Carlo simulated in vivo human brain ^31^P MRS spectra fitting at 7T using OXSA and different algorithms implemented in pyAMARES. (**A**) Ground truth for spectra simulation with slightly perturbed parameters. Gaussian noise is omitted for clarity. (**B**) Relative bias of peak amplitude quantification compared to ground truth. (**C**) CRLB of fitted amplitude for each peak, with the 20% threshold indicated by a green dashed line. (**D**) Pearson’s correlation coefficient (R) between OXSA and pyAMARES quantified amplitudes. Abbreviations: LM: Levenberg–Marquardt algorithm; TRR: trust region reflective algorithm; Init: Initializer using LM; PCr: phosphocreatine; PE: phosphoethenolamine; GPE: glycerophosphoethanolamine; GPC: glycerophosphocholine; Pi: inorganic phosphate; NAD, nicotinamide adenine dinucleotide; UDPG, uridine diphosphoglucose.

To further assess pyAMARES’s performance in a more realistic scenario, we simulated in vivo human brain ^31^P MRS spectra at 7T with slightly perturbed parameters (Figure 4A). We compared the quantification results obtained using OXSA and different algorithms implemented in pyAMARES, including the LM algorithm, TRR algorithm, and TRR with an LM initializer (TRR+Init). The relative bias of peak amplitude quantification (Figure 4B) and the CRLB of fitted amplitude for each peak (Figure 4C) were evaluated. For most metabolites, pyAMARES showed low relative bias and CRLB, with high Pearson’s correlation coefficients (R ≥ 0.95) compared to OXSA (Figure 4D). However, for low SNR peaks such as NAD and UDPG, the performance of pyAMARES and OXSA was less consistent, with higher relative bias and CRLB values. Among the different algorithms in pyAMARES, TRR+Init generally exhibited the lowest relative bias and CRLB, suggesting that the combination of TRR with an LM initializer may be the most robust approach for fitting in vivo ^31^P MRS spectra at 7T. These results demonstrate pyAMARES’s capability to accurately quantify metabolites in simulated in vivo spectra and its potential to outperform OXSA, particularly when using the TRR+Init algorithm.

### 3.3. Multiprocessing Fitting of Dynamic ^31^P MRS Spectra of Muscle at 3T

**Figure 5:**
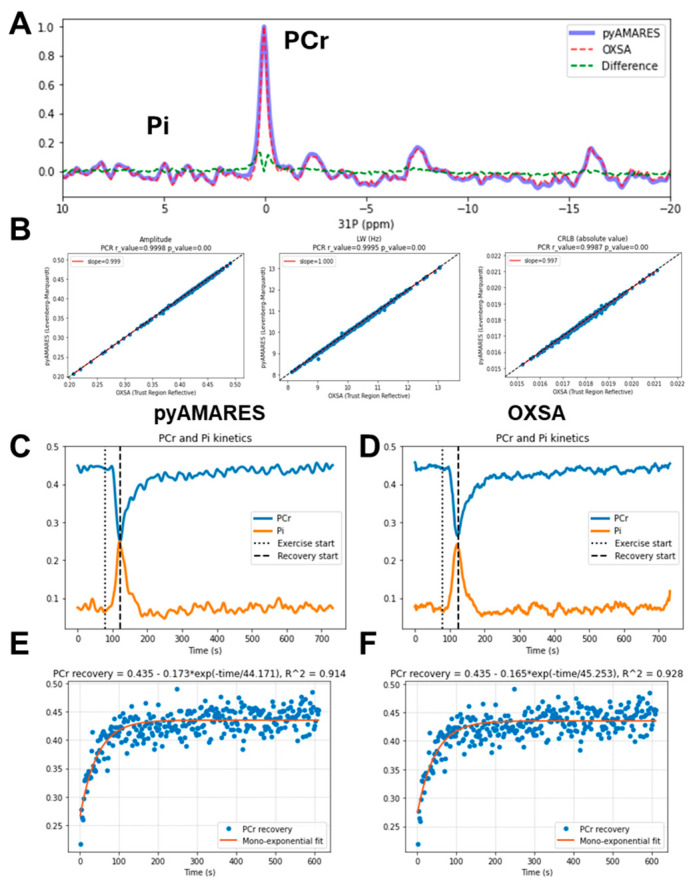
Multiprocessing fitting of dynamic unlocalized ^31^P MRS spectra of the tibialis anterior muscle at 3T using pyAMARES and comparison to OXSA. (**A**). Representative fitting results from pyAMARES (blue solid line) and OXSA (red dash line), with the differences between them shown as green dashed line. The metabolites of interest (PCr and Pi) are labeled. (**B**). Linear correlations between fitted amplitudes (a.u.), linewidths (Hz), and CRLBs obtained by pyAMARES and OXSA. Pearson’s R and the *p*-value for each dataset are shown in the plots. (**C**,**D**) Time courses of PCr (blue) and Pi (orange) amplitudes fitted by pyAMARES (**C**) and OXSA (**D**). The time points at which exercise and recovery start are indicated by dotted and dashed vertical lines, respectively. (**E**,**F**) Mono-exponential fitting of the PCr recovery kinetics using pyAMARES (**E**) and OXSA (**F**). The fitted equations are PC_recover_ = 0.435 − 0.173 × e^−time/44.171^, R^2^ = 0.914 for pyAMARES, and PC_recover_ = 0.435 − 0.165 × e^−time/42.523^, R^2^ = 0.928 for OXSA.

As MRSI and functional MRS/MRSI become more popular, it is often necessary to quantify a large amount of MRS data for numerous voxels or time points. In response, pyAMARES provides the function *run_parallel_fitting_with_progress* for batch fitting that eases the fitting of multi-voxel data with the same fitting prior knowledge set and fitting parameters. Most importantly, the *run_parallel_fitting_with_progress function* uses multiprocessing to circumvent the global interpreter lock (GIL, a mechanism that limits Python to executing only one thread at a time) of Python to enable parallel data fitting. Figure 5 shows an example of using pyAMARES to fit 366 spectra from a dynamic muscle ^31^P MRS dataset and compares the fitting results to those obtained using OXSA, an established MRS quantification software. Figure S3 further demonstrates the efficiency of pyAMARES’s multiprocessing capabilities, showing significant speed improvements when using four or more CPU cores for fitting these 366 spectra.

The representative fitting results (Figure 5A) demonstrate excellent agreement between pyAMARES (blue solid line) and OXSA (red dashed line), with minimal differences (green dashed line) for the metabolites of interest, PCr and Pi. Although subtle discrepancies seem to be present in the PCr peak, linear correlation analyses (Figure 5B) of the fitted amplitudes, linewidths, and CRLBs of the PCr peaks still reveal strong correlations (Pearson’s R > 0.99, *p* < 0.001). The time courses of PCr (blue) and Pi (orange) amplitudes fitted by pyAMARES (Figure 5C) and OXSA (Figure 5D) exhibit identical dynamic patterns (Figure S4), capturing the changes induced by exercise (dotted vertical line) and recovery (dashed vertical line). Mono-exponential fitting of the PCr recovery kinetics (Figure 5E,F) yields nearly identical time constants and coefficients of determination (R^2^) for both pyAMARES (44.171 s, R^2^ = 0.914) and OXSA (42.523 s, R^2^ = 0.928). These results validate the accuracy and reliability of pyAMARES for batch fitting of dynamic MRS data through comparison with the established software OXSA while also demonstrating the utility of the *run_parallel_fitting_with_progress* function for efficient multiprocessing of large datasets.

### 3.4. Versatile MRS Quantification with pyAMARES

PyAMARES is a versatile MRS quantification tool that offers advanced features extending beyond the capabilities of traditional AMARES implementations. These features include the ability to handle spectra with unknown metabolites, perform frequency-selective AMARES [6,22], and eliminate metabolite signals from short-TE MM spectra [17].

Although AMARES is a prior-knowledge-based fitting strategy, it is beneficial to enable the fitting of peaks without known parameters. For example, there may be instances where prior knowledge of added external references for in vivo MRS data is not readily available or when unknown metabolites are present in lesions. The HSVD initializer of pyAMARES, provided as the *HSVDinitializer*, can optimize known fitting parameters. Additionally, this API allows for the generation of fitting parameters from HSVD-decomposed spectra when no prior knowledge of fitting parameters is provided. This enables the fitting of spectra without prior knowledge. As shown in Figure S5A,B, pyAMARES quantifies two peaks in the simulated two-peak spectra using HSVD-generated fitting parameters.

Despite being a time-domain fitting strategy, the frequency-selective version of AMARES allows for the quantification of peaks of interest that are well-separated from nuisance peaks [1]. PyAMARES provides two methods for frequency-selective AMARES as described in the method section. The two-peak example analyzed by both methods yields comparable results (Figure S5C–F). While pyAMARES includes several objective functions, including one with frequency range selection, the objective function API *objective_func* allows users to define their own objective function for fitting the spectra to the AMARES model. This feature provides unparalleled flexibility compared to other AMARES software.

**Figure 6:**
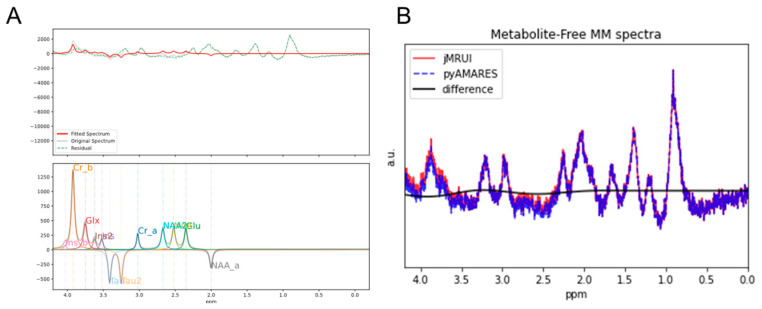
Using AMARES for post-processing: Removal of metabolite residuals from a short echo time (TE) ^1^H MR spectrum at 9.4T. (**A**) Upper panel: Fitting of residual metabolites (red) and the resulting macromolecule (MM) spectrum after subtraction of residual metabolite signals from the original spectrum (green). Lower panel: AMARES modeling of residual metabolite signals. (**B**) Comparison of metabolite-free MM spectra obtained by jMRUI (red) and pyAMARES (blue), showing identical results as confirmed by the flat difference spectrum (black).

Furthermore, pyAMARES demonstrates its usefulness in post-processing applications, such as removing residual metabolite signals from short echo time ^1^H MRS data. This is crucial for accurate quantification of MM contributions, which play a significant role in various physiological and pathological conditions. As shown in Figure 6A, pyAMARES effectively fits and removes residual metabolite signals (red) from a short-TE ^1^H MR spectrum acquired at 9.4T, resulting in a clean MM spectrum (green). The lower panel of Figure 6A displays the AMARES modeling of the residual metabolite signals. Comparing the metabolite-free MM spectra generated by jMRUI (red) and pyAMARES (blue) in Figure 6B reveals virtually identical results, as shown by the flat difference spectrum and quantitatively validated by the strong correlations in fitted parameters (Figure S6). This feature highlights pyAMARES’s potential for integration into existing post-processing pipelines and its value in investigating the role of macromolecules in biological processes.

## 4. Discussion

### 4.1. Validation and Benchmark of pyAMARES

PyAMARES was validated against the established AMARES software using identical data and prior knowledge. By default, pyAMARES employs the LM algorithm to optimize the fitting procedures prior to fitting with the TRR algorithm. Since OXSA was already rigorously validated against the most popular software jMRUI [5], most of the validation of pyAMARES was performed by comparing it to OXSA, as it is easier to compare two cross-platform open-source software packages. The data used for validation and benchmarking include both simulated and in vivo data, with primary comparisons against OXSA (Figure 3, Figure 4 and Figure 5) and additional validation against jMRUI (Figure 6). Overall, pyAMARES obtains nearly identical fitting results as OXSA and jMRUI, suggesting a robust AMARES fitting kernel of pyAMARES.

As a fitting strategy, a good initial estimation of fitting parameters is critical for AMARES to avoid unwanted local minima. The robustness of fitting data that deviates from prior knowledge sets was tested using Monte Carlo simulations at different SNR levels. Notably, pyAMARES is very resilient to non-ideal conditions when initial fitting parameters are significantly off (Figure 3A), especially when SNR ≥ 10. In contrast, while Matlab-based OXSA provides reliable results at SNR ≥ 5 as indicated by a CRLB < 20%, its amplitude error remains ~+/−50%, even at high SNR (SNR = 20), which is substantially larger than pyAMARES. This discrepancy could be caused by the different fitting parameter initialization procedures of OXSA and pyAMARES. OXSA optimizes its initial conditions by solving the linear least square problems for a Lorentzian while only varying amplitude and phases. Thus, it does not address the perturbed chemical shifts in the simulated data (+/−100 Hz in Figure 3). In contrast, pyAMARES uses a LM fitting as an initializer by default, successfully correcting all the perturbed parameters, including the deviated chemical shifts.

The flexibility feature of pyAMARES is exemplified by allowing users to choose their own fitting strategies or even customize minimization objective functions. By default, pyAMARES uses an LM initializer and TRR fitting strategy, while other options such as TRR with or without an LM initializer can also be used. Their performance is benchmarked and compared to OXSA using 3000 simulated in vivo human brain ^31^P MRS data (Figure 4) with slightly perturbed parameters (<5%). The default fitting strategy of pyAMARES (TRR+Init) is the preferred one and is comparable to OXSA.

The excellent agreement between OXSA and pyAMARES is also demonstrated in the dynamic ^31^P MRS spectra acquired at 3T (Figure 5). Not only are the fitted time courses of exercise from the dynamic MRS data nearly identical, but also the amplitude, linewidth, and CRLB are consistent across all time points.

Despite being a quantification strategy, AMARES can also be used as a postprocessing tool. Simicic et al. published a work employing AMARES to fit metabolite residuals in MM spectra. We converted the basis set they published from jMRUI to AMARES. The pyAMARES-obtained metabolite-free MM spectra are identical to those processed by jMRUI (v 7.0) [17].

The comprehensive validation of pyAMARES, encompassing simulated data, in vivo dynamic data fitting, and residual metabolite removal, establishes its reliability, precision, and accuracy as an MRS quantification tool. The software’s robust performance across diverse datasets and its consistency with established software like OXSA and jMRUI build confidence in its application to real-world research and clinical scenarios.

### 4.2. A Flexible, User-Friendly, and Versatile MRS Fitting Tool

The development of pyAMARES bridges the existing software gap between the deep learning-dominant Python ecosystem and the mainstream MRS quantification algorithm AMARES. As a free, open-source Python package, pyAMARES aims to offer flexibility, user-friendliness, and versatility.

PyAMARES can function as a standalone program or a third-party library, seamlessly adapting to various research workflows. Its cross-platform compatibility allows users to run the software on local PCs, web-based platforms like Google Colab, and remote Linux servers, ensuring accessibility for MRI vendors equipped with different platforms and researchers in diverse computing environments.

With a one-line installation process and intuitive operation, pyAMARES allows users to edit parameters using familiar spreadsheet software like Microsoft Excel or OpenOffice, streamlining the I/O of prior knowledge, constraints, and fitting results. This user-friendly feature significantly reduces the learning curve and simplifies the use of the software.

One of the key advantages of pyAMARES is that it allows users to easily fix or fit any parameters according to their specific requirements. The software also supports the use of mathematical expressions to constrain parameters, making it easy to constrain the J-coupled multiplet peaks or two metabolites with a known ratio. Advanced features such as user-defined objective functions and convergence thresholds further enhance pyAMARES’s flexibility, enabling researchers to fine-tune their fitting parameters for optimal results. To our knowledge, compared to other AMARES software, pyAMARES is unique in allowing users to customize the objective function for fitting. Such exclusive advantages enable users to handle unforeseen non-ideal spectra, such as those with non-Gaussian noise or peaks with large deviations.

In terms of versatility, pyAMARES offers features beyond conventional AMARES fitting. It can fit MRS data in the absence of prior knowledge and supports frequency-selective AMARES fitting by either extracting the spectral regions of interest or fitting with an objective function with a selected spectral range (Figure S5).

### 4.3. Enhancing Reproducibility in MR Spectroscopy Research

PyAMARES is designed to enhance reproducibility in MR spectroscopy research. The codebase and comprehensive documentation are freely available via GitHub (https://github.com/HawkMRS/pyAMARES, (accessed on 6 October 2024)) and Read the Docs documentation hosting platform (https://pyamares.readthedocs.io/, (accessed on 6 October 2024)), allowing users to inspect and adapt the software to their specific needs. The PyPI code distribution mechanism (https://pypi.org/project/pyAMARES/, (accessed on 6 October 2024)) enables a one-line installation for pyAMARES. This open-source approach ensures that the AMARES fitting steps are well-defined and can be easily reproduced by other researchers.

A key feature of pyAMARES is the extensive collection of Jupyter Notebook tutorials that guide users through various AMARES fitting strategies step by step. These interactive notebooks combine pyAMARES code, explanatory text, data visualizations, and formatted spreadsheets with reliable fitting highlighted, making it easy for users to understand and modify the fitting process without requiring in-depth expertise in MRS or scientific programming. By providing these tutorials on cloud-based platforms such as Google Colab (https://colab.research.google.com/, (accessed on 6 October 2024)) or Binder (https://mybinder.org/, (accessed on 6 October 2024)), pyAMARES eliminates the need for local software installation and setup, allowing researchers to reproduce pyAMARES examples or explore their own AMARES fitting in a web browser.

The development of pyAMARES benefits from the open-source community. It relies on open-source Python libraries such as lmfit [35] for fitting and spec2nii for vendor-specific data reading. PyAMARES is validated against the open-source AMARES software OXSA, and its metabolite residual modeling in MM spectra function is verified using published results from Simicic et al. [17]. These collaborations and validations within the open-source community enhance the reliability and credibility of pyAMARES.

The permissive Berkeley Software Distribution (BSD) license of pyAMARES and the detailed tutorials promote a more inclusive and collaborative research environment. Researchers can request new features, contribute improvements, and build upon their own MRS analysis pipelines that depend on pyAMARES.

In summary, pyAMARES serves as a powerful tool for reproducible research in MR spectroscopy. By embracing open-source principles, providing comprehensive documentation, and offering accessible, cloud-based tutorials, pyAMARES empowers researchers to conduct transparent, replicable, and collaborative studies, ultimately advancing the field of MRS and its applications in biomedical research.

### 4.4. Limitations

While pyAMARES demonstrates flexible and robust performance in quantifying both simulated and in vivo MRS data, there are some limitations to consider.

Although AMARES can quantify J-coupled metabolites in ^1^H MRS [8], incorporating J-coupling information for numerous metabolites into the prior knowledge is challenging in practice. For ^1^H MRS quantification, other software packages like LCModel are often preferred because they can easily include J-coupling information within their basis sets. However, AMARES remains valuable for specific ^1^H MRS applications, such as removing residual metabolite signals in short-TE spectra [17]. AMARES may also be useful for quantifying long-TE ^1^H MRS data, where the spectrum typically contains fewer major peaks with minimal baseline signals [36].

In addition to the scientific considerations, pyAMARES also faces some technical limitations: It offers unique flexibility, particularly allowing users to fix and fit given parameters. However, this flexibility comes at a cost: unlike OXSA, pyAMARES currently does not support passing analytical Jacobian to its non-linear least squares fitting and must rely on numerical differentiation, which affects its computational speed. Another potential limitation is the GIL in Python, which can restrict the performance of multi-threaded computing. Although the quantification of multivoxel MRS, such as MRSI or dynamic MRS, are I/O-bound tasks and can therefore be accelerated by the multiprocessing API of pyAMARES (Figure S3), the GIL may still impact the overall speed of the software, especially when performing complex analyses compared to OXSA and jMRUI. However, future releases of Python may address this limitation, and the performance of pyAMARES can benefit from the multithreading powered by lower-level libraries at the C level, such as OpenBLAS and Intel MKL, depending on the deployment of the Python environment of specific users.

PyAMARES is distributed as a Python library, which takes advantage of the powerful Python ecosystem but may bring some restrictions that are not necessarily limitations. First, compared to the Java and MATLAB implementations of AMARES, pyAMARES relies on external libraries to read vendor-specific MRS data. Thus, users should carefully extract correct spectral parameters from their vendor-specific data, such as the dead time between the RF excitation and the FID acquisition, as pyAMARES does not handle the parameter parsing step. Moreover, it is not within pyAMARES’s scope to reconstruct and post-process the MRS spectrum. PyAMARES does not support routine preprocessing operations such as coil combination, apodization, zero-filling, or eddy-current correction because numerous existing tools (jMRUI [21], FID-A [37], MRSpa [38], Osprey [39], TARQUIN [40], etc.) are already well-equipped for these tasks. Moreover, some of these tools are Python-based or have a Python API (e.g., FSL-MRS [23], SUSPECT [25], VESPA [24]) and can easily work together with pyAMARES. Thus, we do not consider the lack of built-in reconstruction and preprocessing a real limitation.

Similarly, pyAMARES does not implement a graphical user interface (GUI), which some users may prefer for a more intuitive workflow. However, pyAMARES is designed to be used in the background as a command-line tool, which can be called by an online reconstruction tool on the scanner or offline batch MRS analysis tools on a computing server. As a Python library, users can utilize pyAMARES within Jupyter notebooks for a decent interactive interface, mitigating the need for a dedicated GUI.

## 5. Conclusions

In conclusion, pyAMARES represents a valuable addition to the arsenal of MRS quantification tools, offering a flexible, user-friendly, and versatile solution for researchers and clinicians. By implementing the AMARES algorithm in Python, pyAMARES bridges the gap between deep learning frameworks and traditional MRS fitting methods. Its robust performance, validated against established software and across diverse datasets, demonstrates its reliability for real-world applications. The open-source nature of pyAMARES and its comprehensive documentation and interactive tutorials promote reproducibility and collaboration in MR research. While some limitations exist, such as single-tasking and performance restrictions due to Python’s GIL, the software’s integration with the Python ecosystem and compatibility with existing MRS analysis tools make it a valuable contribution to the field.

As MRS continues to play a crucial role in studying metabolism and disease, pyAMARES provides researchers with a powerful tool for accurate, efficient, and reproducible quantification. Its permissive open-source license invites community contributions and improvements, fostering a more inclusive and collaborative research environment. With pyAMARES, researchers can focus on advancing MRS applications and biomedical discoveries, confident in the reliability and transparency of their quantification pipeline.

## Figures and Tables

**Table 1:** Prior knowledge dataset for in vivo ^31^P MRS of human brain at 7T. This table shows the initial values and constraints for fitting metabolite peaks using pyAMARES. The upper half defines initial values for amplitude, chemical shift, linewidth, phase, and lineshape (g) for each metabolite. The lower half specifies the fitting constraints. Metabolite peaks are organized in columns, showing βATP and αATP multiplets, with J-coupling splitted multiplets (e.g., BATP, BATP2, BATP3) grouped together. Values are in arbitrary units for amplitude and g, ppm for chemical shift, Hz for linewidth, and degrees for phase. Constraints are set using brackets, with (min, max) indicating ranges and single values denoting fixed parameters. When specifying only one bound, using a single bound with an open bracket defines only one limit. For example, (0, means the value must be greater than 0 with no upper limit, while, 180) means the value must be less than 180 with no lower limit. Mathematical expressions (e.g., BATP/2, BATP-15Hz) are used to relate parameters of multiplet peaks. Lines starting with # are comments and are ignored during processing. Data adapted from Ren et al. [28]. For detailed documentation on the format of the prior knowledge dataset and the complete version of Table 1, refer to https://pyamares.readthedocs.io/en/latest/notebooks/priorknowledge.html (accessed on 6 October 2024).

	A	B	C	D	E	F
**1**	# Ren et al. [28]					
**2**	Index	BATP	BATP2	BATP3	AATP	AATP2
**3**	Initial Values					
**4**	amplitude	1.41	BATP/2	BATP/2	1.545	AATP
**5**	chemicalshift	−16.15	BATP-15Hz	BATP+15Hz	−7.49	AATP-16Hz
**6**	linewidth	58.12	BATP	BATP	32.28	AATP
**7**	phase	0	BATP	BATP	BATP	BATP
**8**	g	0	0	0	0	0
**9**	Bounds					
**10**	amplitude	(0,	(0,	(0,	(0,	(0,
**11**	chemicalshift	(−16.30, −16.00)	(−16.30, −16.00)	(−16.30, −16.00)	(−7.72, −7.42)	(−7.72, −7.42)
**12**	linewidth	(54.335, 61.911)	(54.335, 61.911)	(54.335, 61.911)	(31.226, 33.327)	(31.226, 33.327)
**13**	phase	(−180, 180)	(−180, 180)	(−180, 180)	(−180, 180)	(−180, 180)
**14**	g	(0, 1)	(0, 1)	(0, 1)	(0, 1)	(0, 1)
**15**	# Use the same phase for all peaks					

## Data Availability

The pyAMARES software and its source code are openly available on GitHub (https://github.com/HawkMRS/pyAMARES, (accessed on 6 October 2024)). Detailed documentation, including tutorials and usage examples, is accessible at https://pyamares.readthedocs.io/, (accessed on 6 October 2024). The source code and datasets used for validation and benchmarking in this study are available in the pyAMARES GitHub repository. The dynamic ^31^P MRS spectra of muscle are available upon request from M.V. All jMRUI files required for modeling residual metabolites in MM spectra can be accessed as supplementary information (SI) of Simicic et al. [17], under a CC BY-NC-ND 4.0 license.

## Supplementary Materials

The following supporting information can be downloaded at: https://www.mdpi.com/article/10.3390/diagnostics14232668/s1, Figure S1. Editable fitting parameter *dataframe* in pyAMARES; Figure S2. Prior knowledge datasets and fitting results; Figure S3: Four or more CPU cores considerably boost the computing speed of pyAMARES; Figure S4. Overlay of PCr and Pi time courses fitted by pyAMARES and OXSA; Figure S5: Versatile MRS quantification capabilities of pyAMARES; Figure S6. Comparison of residual metabolite modeling between pyAMARES and jMRUI.
