# Supplementary material for: PyAMARES, an Open-Source Python Library for Fitting Magnetic Resonance Spectroscopy Data
Source: Diagnostics (Basel). 2024 Nov 27;14(23):2668. doi: 10.3390/diagnostics14232668 (PMC11639817; doi:10.3390/diagnostics14232668)
Supplement: Supplementary file 1 [file diagnostics-14-02668-s001.zip › diagnostics-3282832-supplementary.pdf]

SUPPORTING INFORMATION FOR

PyAMARES, an Open-Source Python Library for Fitting Magnetic Resonance Spectroscopy Data

```
multiplet_obj.initialParams
```

name	value	initial value	min	max	vary	expression
ak_BATP	2.82000000	2.82	0.00000000	inf	True	
freq_BATP	-1944.00000	-1963.1999999999998	-1944.00000	-1932.00000	True	
dk_BATP	173.120605	173.12060476871915	0.00000000	inf	True	
phi_BATP	0.00000000	0.0	-3.14159265	3.14159265	True	
g_BATP	0.00000000	0.0	0.00000000	1.00000000	False	
ak_BATP2	1.41000000	1.41	0.00000000	inf	False	ak_BATP/2
freq_BATP2	-1944.00000	-3763.2	-1944.00000	-1932.00000	False	freq_BATP-15
dk_BATP2	173.120605	173.12060476871915	0.00000000	inf	False	dk_BATP
phi_BATP2	0.00000000	0.0	-3.14159265	3.14159265	False	phi_BATP

Figure S1: Editable fitting parameter dataframe in pyAMARES. The *vary* column allows users to specify whether a given parameter should be fitted or fixed. The *expression* column enables the constraint of one parameter to another using mathematical expressions. This dataframe can be manipulated directly using Python's Pandas library or through CSV files using the *save_parameter_to_csv* and *load_parameter_from_csv* APIs, providing flexible options for parameter management.

A

	A	B	C	D
1	Index	Gas	Membrane	RBC
2	Initial Values			
3	amplitude	1	0.6	0.2
4	chemicalshift	0	197	210
5	linewidth	40	10	10
6	phase	0	0	Membrane
7	g	0	0.1	0
8	Bounds			
9	amplitude	(0,	(0,	(0,
10	chemicalshift	(-25,25)	(192, 205)	(200,215)
11	linewidth	(0,	(0,200)	(0,200)
12	phase	(-180,180)	(-180,180)	(-180,180)
13	g	(0,1)	(0,1)	(0,1)

B

	A	B	C	D	E
1	Index	DHO	Glucose	Glx	Lactate
2	Initial Values				
3	amplitude	10	1	0.7	0.1
4	chemicalshift	4.7	3.8	2.3	1.3
5	linewidth	10	10	10	10
6	phase	0	DHO	DHO	DHO
7	g	0	0	0	0
8	Bounds				
9	amplitude	(0,	(0,	(0,	(0,
10	chemicalshift	(4.2, 5.2)	(3.3, 4.3)	(1.8,2.8)	(0.8, 1.8)
11	linewidth	(0,	(0,100)	(0,100)	(0, 100)
12	phase	(-180,180)	(-180,180)	(-180,180)	(-180,180)
13	g	(0,1)	(0,1)	(0,1)	(0,1)

C

name	amplitude	chem shift(ppm)	LW(Hz)	phase(deg)	SNR	CRLB(%)
Gas	0.004	0.062	6.114	-44.828	10.185	0.487
Membrane	0.002	196.126	200.000	-39.286	5.340	4.832
RBC	0.001	204.613	199.132	-39.286	2.192	12.405

D

name	amplitude	chem shift(ppm)	LW(Hz)	phase(deg)	SNR	CRLB(%)
DHO	0.004	-0.090	39.680	-19.241	2.361	4.619
Glucose	0.001	-1.052	30.109	-19.241	0.682	13.969
Glx	0.002	-2.571	60.791	-19.241	1.088	19.561
Lactate	0.001	-3.733	100.000	-19.241	0.476	54.702

Figure S2. Prior knowledge datasets and fitting results for (A,C) a voxel of hyperpolarized ^{129}Xe MRSI acquired from healthy porcine lungs at 3T showing membrane and red blood cells (RBC) peaks; (B, D), a voxel of in vivo brain ^{2}H MRSI acquired at 3T showing DHO and other peaks. In the fitting results tables (C, D), green rows indicate reliable fits with CRLB < 20% and red rows (such as lactate) indicate less reliable fits with CRLB > 20%. Abbreviations: RBC, red blood cells; DHO, deuterated water; Glx, combined signals of glutamate and glutamine.

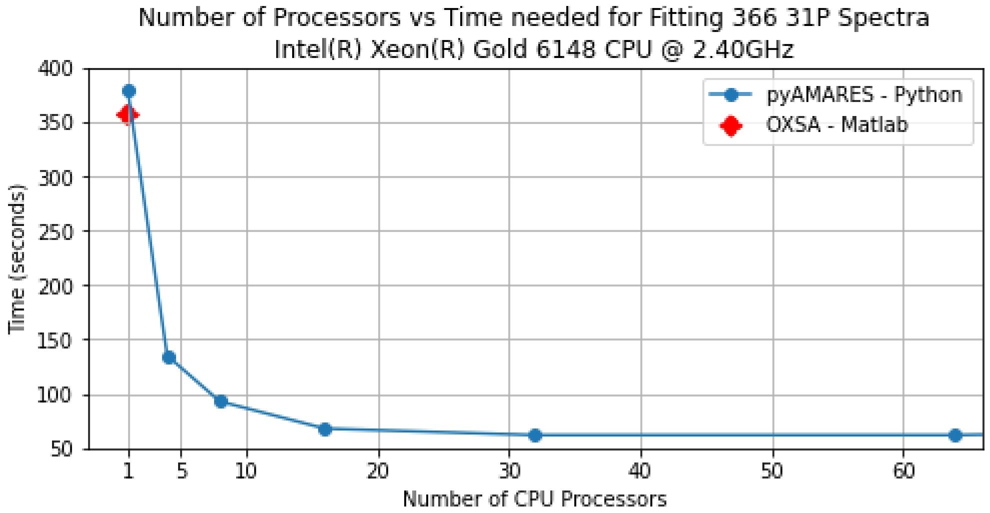

Figure S3: 4 or more CPU cores considerably boost the computing speed of pyAMARES. The processing time versus the number cores of CPU of pyAMARES and OXSA were benchmarked on a Linux platform with Intel(R) Xeon(R) Gold 6148 CPU @ 2.40GHz. PyAMARES (blue line) demonstrates significant speed improvements with increased core count, particularly beyond 4 cores. For comparison, OXSA's (MATLAB-based) default performance is

shown (red diamond). At lower core counts, pyAMARES maintains comparable processing times to OXSA, while offering superior scalability with additional cores.

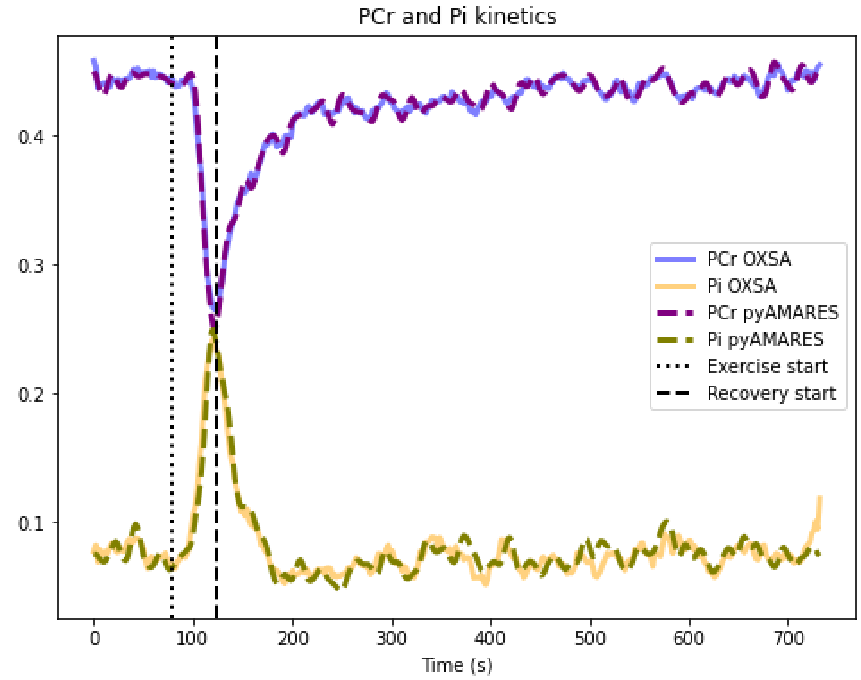

Figure S4. Overlay of PCr and Pi time courses fitted by pyAMARES and OXSA. The perfect overlap between pyAMARES (dashed lines, Figure 5C) and OXSA (semi-transparent lines, Figure 5D) demonstrates the consistency between the two software packages. The exercise start and recovery start times are indicated by dotted and dashed vertical lines, respectively.

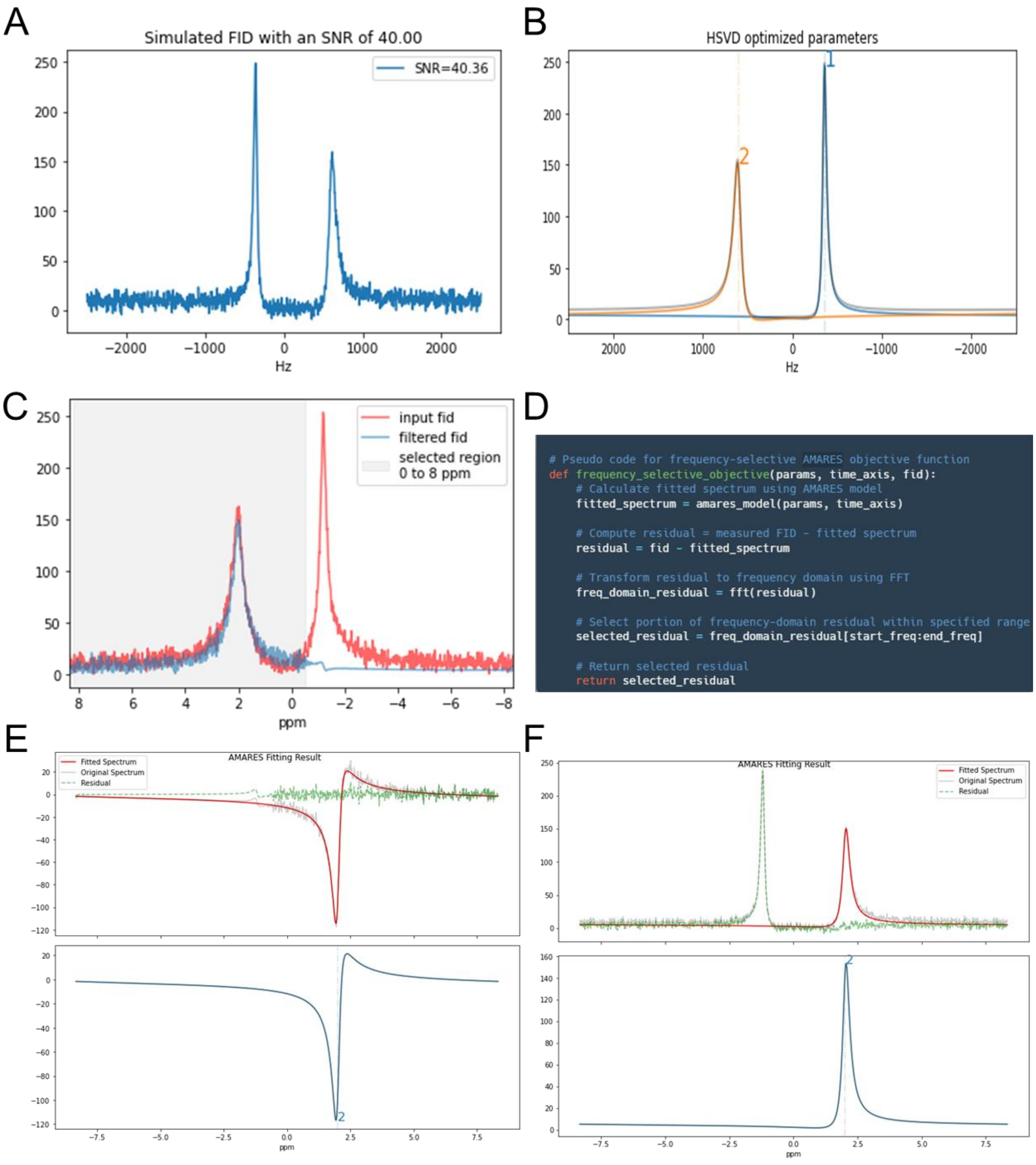

Figure S5. Versatile MRS quantification capabilities of pyAMARES. A) Simulated two-peak FID with an SNR of 40 (Fourier transformed). B) Identification and quantification of two peaks using the *HSVDinitializer* API without prior knowledge. C) Spectrum after applying an MPFIR filter to extract peaks within the 0-8 ppm range. D) Pseudo code of the frequency-selective AMARES objective function. E) Results of frequency-selective AMARES fitting using the MPFIR filter method. F) Results of frequency-selective AMARES fitting using the objective function with a specified spectral range.

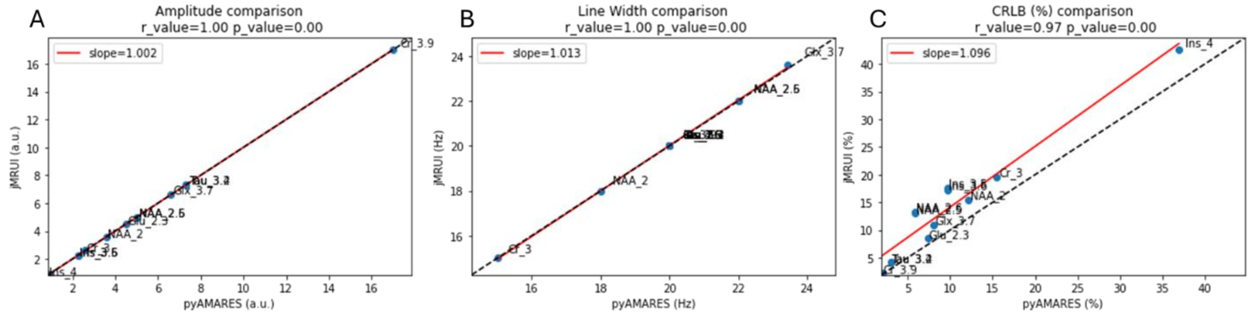

Figure S6. Comparison of residual metabolite modeling between pyAMARES and jMRUI. (A) Correlation of fitted peak amplitudes between pyAMARES (x-axis) and jMRUI (y-axis), showing excellent agreement with a slope of 1.002 (r=1.00, p<0.001). (B) Comparison of fitted linewidths demonstrates a strong correlation between the two software packages (slope=1.013, r=1.00, p<0.001). (C) CRLB from both methods show excellent agreement (slope=1.096, r=0.97, p<0.001). In all panels, the red line represents the linear fit, while the dashed black line indicates the identity line (slope=1). The strong correlations and slopes close to unity across all parameters demonstrate that pyAMARES produces results highly consistent with jMRUI for metabolite residual modeling in macromolecule spectra.
